# Root-to-shoot signaling positively mediates source-sink relation in late growth stages in diploid and tetraploid wheat

**DOI:** 10.1186/s12870-024-05046-z

**Published:** 2024-06-03

**Authors:** Asfa Batool, Shi-Sheng Li, Dong-Xia Yue, Fazal Ullah, Ling Zhao, Zheng-Guo Cheng, Chao Wang, Hai-Xia Duan, Guang-Chao Lv, Zeeshan ul Haq, Khalil Ahmed, Yan-Wen Gui, Li Zhu, Yun-Li Xiao, You-Cai Xiong

**Affiliations:** 1 College of Biology and Agricultural Resources, Huanggang Normal University, Huanggang, 438000 China; 2 MOE Key Laboratory of Western China’s Environmental Systems, College of Earth and Environmental Sciences, Lanzhou University, Lanzhou, 730000 China; 3 State Key Laboratory of Herbage Improvement and Grassland Agro-Ecosystems, College of Ecology, Lanzhou University, Lanzhou, 730000 China; 4 Faculty of Agriculture, University of Sargodha, Sargodha, 40100 Pakistan

**Keywords:** Abscisic acid, Non-hydraulic signaling, Source-sink relations, Cytokinin, Primitive wheat, Yield formation

## Abstract

Non-hydraulic root source signaling (nHRS) is a unique positive response to soil drying in the regulation of plant growth and development. However, it is unclear how the nHRS mediates the tradeoff between source and sink at the late growth stages and its adaptive mechanisms in primitive wheat. To address this issue, a root-splitting design was made by inserting solid partition in the middle of the pot culture to induce the occurrence of nHRS using four wheat cultivars (MO1 and MO4, diploid; DM22 and DM31, tetraploid) as materials. Three water treatments were designed as 1) both halves watered (CK), 2) holistic root system watered then droughted (FS), 3) one-half of the root system watered and half droughted (PS). FS and PS were designed to compare the role of the full root system and split root system to induce nHRS. Leaves samples were collected during booting and anthesis to compare the role of nHRS at both growth stages. The data indicated that under PS treatment, ABA concentration was significantly higher than FS and CK, demonstrating the induction of nHRS in split root design and nHRS decreased cytokinin (ZR) levels, particularly in the PS treatment. Soluble sugar and proline accumulation were higher in the anthesis stage as compared to the booting stage. POD activity was higher at anthesis, while CAT was higher at the booting stage. Increased ABA (nHRS) correlated with source-sink relationships and metabolic rate (i.e., leaf) connecting other stress signals. Biomass density showed superior resource acquisition and utilization capabilities in both FS and PS treatment as compared to CK in all plants. Our findings indicate that nHRS-induced alterations in phytohormones and their effect on source-sink relations were allied with the growth stages in primitive wheat.

**Supplementary Information:**

The online version contains supplementary material available at 10.1186/s12870-024-05046-z.

## Background

Water stress is considered one of the major limiting factors for crop production worldwide. Various studies have been performed to understand the plant responses and adaptive strategies to drought. Roots can sense and respond to the soil moisture contents by transferring chemical signals to the above plant parts without changing the water status [1]. Stomatal behaviour is one of the most sensitive indicators from a plant to drying soil. Simultaneously, a sequence of defensive responses might be elicited, such as stomatal closure, reduction of leaf expansion rate, and osmolytes production [2, 3], which lead to the reduction in water loss.

According to root to shoot communication theory [4], there are two types of root-sourced signals: one is nHRS (non-hydraulic root source signals, i.e., chemical signals) while the other one is HRS (hydraulic root source signals). Investigations concerning root-sourced signals until now have been principally on the nature of the soil-drying signals. Various ‘split-root’ experiments have been conducted so far to reveal the objective presence of nHRS in plants. The performance of different species in directing root-sourced signals might have a key effect on plant resistance to drought adaptation.

Abscisic acid (ABA), a phytohormone, is advocated as one of the major signals in the long-distance root-to-shoot signaling process [5, 6]. ABA is taken as one of the root-operated hormones produced by the root [7] transported to the shoot that regulates the stomatal behaviour to soil drying [8]. Whereas the reduced correlation between stomatal conductance and Xylem ABA, but the positive correlation with the leaf ABA depicts that regulation of stomatal closure in response to drying soil is allied with the ABA accumulation in leaf tissues of plants [9, 10]. Cytokinin is another plant hormone that can induce stomatal opening in plants [11]. Synthetic and endogenous cytokinins might counteract and induce stomatal closure in maize leaves [12]. Cytokinins can counteract the process of ABA-induced stomatal closure and show potential crosstalk for ABA signaling to guard cells and exhibit antagonistic effects [13, 14].

Plants’ early response to drought stress during drying soil is mainly allied with survival and adaptation to adverse conditions [15]. An increase in soil drying can cause the enhancement of reactive oxygen species (ROS) production and antioxidant defense activities [16, 17]. Early triggering of non-hydraulic root source chemical signals (nHRS) acts as a typical “early-warning” response to drought. It can maintain the homeostasis between ROS and antioxidant defense that regulates metabolism under water deficit. In many cases, studies suggest that ROS production is time-dependent and induced by chemical signals perception [18]. ROS production might act as a secondary messenger allied with the growth of plant and yield production [19]. Reduced levels of ROS, such as hydroxyl radicals (^.^OH), hydrogen peroxide (H_2_O_2_), and superoxide anion radicals (O_2_^−^), plus the complex upregulation of antioxidant enzymatic and non-enzymatic compounds activity downstream the metabolism effects. Root signal-operated compounds increase the activity of ascorbate peroxidase (APX), catalases (CAT), superoxide dismutases (SOD), and glutathione reductase (GR); all are considered as key antioxidant enzymes to prevent oxidative damage under various abiotic stresses [20].

Wheat has different progenitors and is divided into three levels of ploidy. Cultivated wheat occurs at all three ploidy levels; however, wild wheat is present at diploid and tetraploid levels [15]. Different physiological changes and potential processes have been accompanied during the evolution of wheat [21, 22]. Wide genetic diversity of the wheat gene pool has been stored in the ex-situ gene banks. A and B genomes for MO1 and MO4 (*Triticum monococcum* L.), DM22, and DM31 (*Triticum dicoccum* Schuebl L.) have been recognized as donors of modern hexaploid (*Triticum aestivum* L.) wheat genome having (AABBDD, 6n = 42) [23]. Wheat, the most widely grown cereal crop in the Poaceae family, accounts for nearly 30% of global grain production and 50% of global grain trade. According to FAO estimates, an additional 198 million tonnes of wheat will be needed by 2050 to meet global demand, meaning that wheat production in developing nations must increase by 77% [24]. Plant physiology and biochemistry are significantly altered by drought. In response to water stress, plants exhibit a variety of morphological changes; nevertheless, under extreme stress conditions, plants experience functional impairment as well as the loss of entire plant portions. The crucial times for wheat water requirements are jointing, tillering, and anthesis [25]. Water is necessary for wheat growth at every stage, however during key stages, wheat is more susceptible to water scarcity, and any reduction in water availability during these stages results in a large yield loss. Plant development, phenology, respiration, photosynthesis, and assimilate partitioning are all impacted by drought stress [26].

The source-sink relationship is an important physiological process in crops to determine dry matter accumulation. Molecular mechanisms regulate plants’ source-sink balances, including phytohormones, genes, and metabolites [27]. Metabolites are the final products of cellular processes, providing a snapshot of a plant’s biological status at a given developmental stage under different environmental conditions [28]. Different metabolomic and transcriptomic analyses showed that sugar metabolism is the crucial metabolic and transcriptional component differentiating floral organ susceptibility or tolerance to stress. The source has the potential for photosynthesis, and the sink is the potential capacity to metabolize or store photosynthetic products [29]. In wheat, the leaf is the main source from which the grains, sheath, stem, and other plant parts are formed. The sink refers to the new tissues and grains further differentiated into metabolic and storage sinks [30]. Grain is the main component of the sink in the late growth period of wheat. Therefore, source (leaves photosynthetic activity) and sink (grains storage ability) are the main factors that could limit wheat grain yield under drought [31]. Under soil drying, the interaction between source and sink is very important as it could affect crop yield formation [32]. For example, when the sink capacity is small, crops may not be achieved at their potential level. Therefore, it is important to study the sink-source relationship under nHRS in primitive wheat.

Source-sink relations in plants under drought are one of the emerging issues with the recent advancement in scientific approaches. A broad understanding of source-sink manipulation, metabolic dynamics, and the relationship between -induced phytohormones such as ABA and ZR and their signaling crosstalk with other stress signals at different growth stages in primitive wheat species has not been discussed in previous studies. This study will reveal the correlation between root-to-shoot signaling and the changes in source-sink relationship in late growth stages in diploid and tetraploid wheat in order to provide some theoretical support for future breeding programs. Therefore, the major objective of this study was to elucidate the physiological and metabolic responses of primitive wheat (*T. monococcum & T. dicoccum*) to nHRS at the booting (vegetative stage) and flowering stage (a critical stage affecting grain yield). The influence of metabolic rate connecting other stress signals on wheat productivity and yield formation, which is critical for selecting germplasm, is also studied. Our findings provide comprehensive information on physiological and metabolic dynamics associated with drought stress tolerance in primitive wheat. This would be a major step for accelerating the development of wheat-tolerant varieties using biomarker-assisted selection.

## Results

### nHRS enhanced drought-induced ABA accumulation and decreased CKs production in different ploidy wheat accessions

**Fig. 1:**
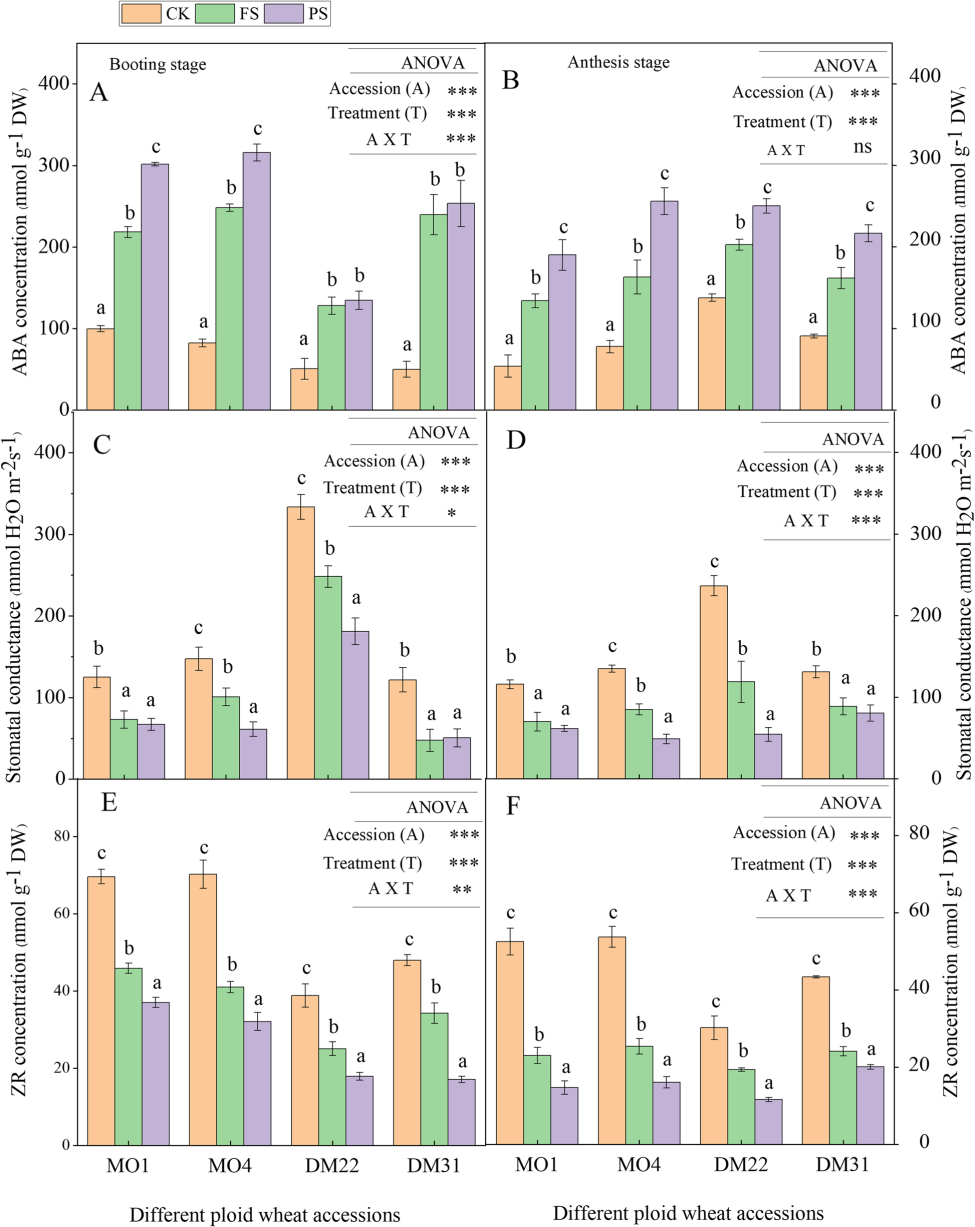
**A**-**F** Dynamics of leaf ABA (abscisic acid) and ZR (zeatin riboside) concentrations and stomatal conductance under the operation of nHRS in diploid and tetraploid wheat at booting and anthesis stages. Leaf ABA concentration at bootig stage (**A**), leaf ABA concentration at anthesis stage (**B**), stomatal conductance at booting stage (**C**), stomatal conductance at anthesis stage (**D**), ZR concentration at booting stage (**E**), and ZR concentration at anthesis stage (**F**), in diploid and tetraploid wheat species in response to nHRS (non-hydraulic root source signaling) conditions

nHRS significantly (*P* < 0.05) enhanced ABA accumulation in all species’ PS plants at both booting and anthesis developmental stages. At the booting and anthesis stages of *T. monococcum* and *T. dicoccum*, respectively, the maximum accumulation in average relative ABA was to approximately 40% and 60% of FS controls and to approximately 30% and 50% of FS controls, respectively (Fig. 1A, B). Higher ABA concentration was linked with the decline in stomatal conductance (*gs*), and it mostly remained low for diploid and tetraploid primitive wheat species. Although MO1 and DM31 did not have a significant difference between FS and PS and showed a similar trend at both growth stages (Fig. 1C, D). ABA concentration of FS controls was significantly enhanced compared to those of the CK controls across all wheat accessions, which caused a decline in *gs* of all FS individuals.

Cytokinin (zeatin) concentration was reduced in both FS and PS plants to those of the CK controls in all wheat species. The ZR concentration of PS plants to its FS controls was about 39% in *T. dicoccum* and 37% in *T. monococcum* at booting and anthesis, respectively (Fig. 1E, F). These results suggest that ZR enhances non-hydraulic ABA-induced stomatal closure more in diploid species than tetraploid species.

### nHRS maintained the Leaf water status in all wheat species and nHRS reduced gas exchange characteristics at both growth stages

**Fig. 2:**
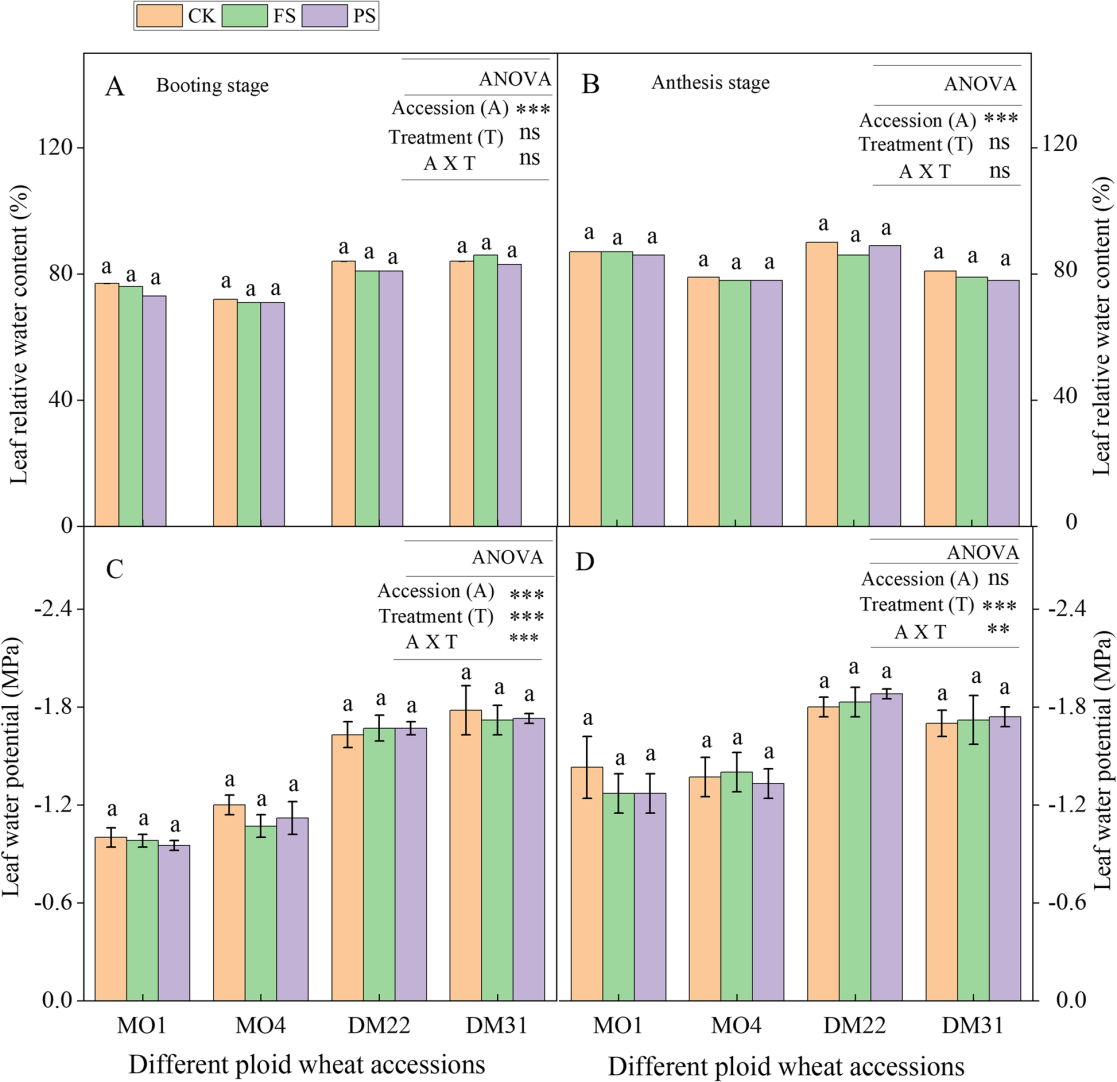
**A**-**D** Leaf water status under the operation of nHRS in different ploidy (diploid, tetraploid) wheat accession at booting and anthesis stage. Leaf water content at booting stage (**A**), leaf relative water content at anthesis stage (**B**), leaf water potential at booting stage (**C**), and leaf water potential at anthesis stage (**D**), in diploid and tetraploid wheat species in response to nHRS (non-hydraulic root source signaling) conditions

Leaf relative water content (LRWC) of PS and FS was similar to those of the CK controls in all species during the booting and anthesis period (Fig. 2A, B). Leaf water potential (LWP) of PS and FS plants was mostly similar to those of the CK controls under drought stress (Fig. 2C, D). However, the LWP of tetraploid wheat species was higher than diploid species at both the booting and anthesis stages. These results indicate that higher ABA accumulation in half dried root system triggered the root to shoot, signaling cross-talk and increasing the plant leaf water potential and maintained as CK (Fig. 2).

The transpiration rate significantly declined in PS plants at booting and anthesis, while there was no significant difference between PS and FS in MO1 and DM31 at both growth stages (Table 1). The photosynthetic rate was significantly (*P* < 0.05) reduced in PS plants than of its FS controls across all accessions at both developmental stages except DM31, which did not show a significant difference between FS and PS treatments at both booting and anthesis stages, respectively. The interaction between accession and treatment had a significant effect. Reduction in photosynthetic and transpiration rate indicates the role of ABA as an nHRS signal to induce stomatal closure. Instant water use efficiency significantly (*P* < 0.05) increased in FS in MO1 and DM31 plants and PS in MO4 and DM22 plants at the booting stage. While at anthesis, it was significantly increased in FS in MO1 and FS and PS in DM22, respectively (Table 1).

**Table 1:** Responses of leaf gas exchange and WUE-related parameters to nHRS in primitive wheat at booting. Anthesis and maturity stages

		Photosynthetic rate		Transpiration rate		Instant water use efficiency		Grain yield (g/plant)	Water consumption (kg/plant)	WUE_G_	WUE_AGB_
Cultivars	Treatment	BS	AS	BS	AS	BS	AS				
	CK	21.33 ± 0.69c	19.93 ± 0.62c	3.93 ± 0.06b	3.46 ± 0.06b	6.27 ± 0.16b	5.75 ± 0.15b	0.98 ± 0.11b	2.51 ± 0.13b	0.39 ± 0.06a	1.32 ± 0.19a
MO1	FS	18.87 ± 0.88b	15.57 ± 0.37b	2.37 ± 0.13a	2.13 ± 0.06a	8.01 ± 0.11c	7.37 ± 0.08c	0.79 ± 0.07ab	2.15 ± 0.05a	0.37 ± 0.04a	1.36 ± 0.12a
	PS	11.05 ± 0.25a	11.06 ± 0.25a	2.25 ± 0.09a	2.25 ± 0.09a	4.97 ± 0.08a	4.97 ± 0.08a	0.66 ± 0.08a	1.99 ± 0.06a	0.33 ± 0.02a	1.23 + 0.06a
	CK	23.07 ± 0.41c	19.27 ± 0.62c	4.35 ± 0.05c	3.99 ± 0.05c	5.34 ± 0.14a	4.85 ± 0.11a	1.07 ± 0.11b	2.48 ± 0.09c	0.43 ± 0.06a	1.53 ± 0.19a
MO4	FS	18.16 ± 0.36b	15.61 ± 0.17b	3.11 ± 0.11b	3.21 ± 0.11b	5.92 ± 0.13a	4.98 ± 0.19a	0.85 ± 0.11b	2.08 ± 0.13b	0.40 ± 0.04a	1.48 ± 0.14a
	PS	15.23 ± 0.31a	10.13 ± 0.41a	2.15 ± 0.14a	2.15 ± 0.14a	7.91 ± 0.73b	5.21 ± 0.43a	0.54 ± 0.05a	1.73 ± 0.04a	0.32 ± 0.03a	1.24 + 0.07a
	CK	18.95 ± 0.34c	14.49 ± 0.17c	8.62 ± 0.09c	6.01 ± 0.32c	2.20 ± 0.04a	2.52 ± 0.10a	2.52 ± 0.21b	3.31 ± 0.07c	0.76 ± 0.05a	1.82 ± 0.14a
DM22	FS	15.65 ± 0.24b	11.75 ± 0.30b	6.95 ± 0.11b	4.33 ± 0.26b	2.26 ± 0.03a	2.89 ± 0.15b	2.13 ± 0.16ab	2.39 ± 0.01b	0.89 ± 0.06a	2.06 ± 0.15a
	PS	13.72 ± 0.32a	8.06 ± 0.41a	5.20 ± 0.14a	2.48 ± 0.16a	2.64 ± 0.02b	3.34 ± 0.09c	1.78 ± 0.15a	1.99 ± 0.02a	0.90 ± 0.06a	2.18 + 0.17a
	CK	11.98 ± 0.53b	20.86 ± 0.19b	4.39 ± 0.24b	4.45 ± 0.07b	2.77 ± 0.04a	4.70 ± 0.06a	2.32 ± 0.29b	2.71 ± 0.18b	0.85 ± 0.06a	2.33 ± 0.11a
DM31	FS	7.14 ± 0.18a	15.53 ± 0.49a	1.88 ± 0.27a	3.11 ± 0.08a	8.20 ± 1.89b	4.99 ± 0.05a	1.23 ± 0.14a	1.86 ± 0.08a	0.87 ± 0.09a	1.89 ± 0.26a
	PS	7.03 ± 0.52a	14.8 ± 0.23a	2.61 ± 0.32a	3.13 ± 0.14a	3.10 ± 0.23a	4.88 ± 0.19a	1.33 ± 0.16a	1.68 ± 0.04a	0.88 ± 0.09a	2.26 + 0.27a
Accession (A)		***	***	***	***	***	***	***	***	***	***
Treatment (T)		***	***	***	***	***	**	***	***	NS	NS
V × T		*	**	NS	***	NS	**	NS	***	NS	NS

Values are represented as means ± S. E. of the mean. Different letters within one cultivar for three different treatments at *P* < 0.05

*BS* Booting stage, *AS* Anthesis stage, *CK* Well water, *FS* Full root zone water stress, *PS* Partial root zone water stress

### nHRS reduced the reactive oxygen species and increased proline content and soluble sugar

**Fig. 3:**
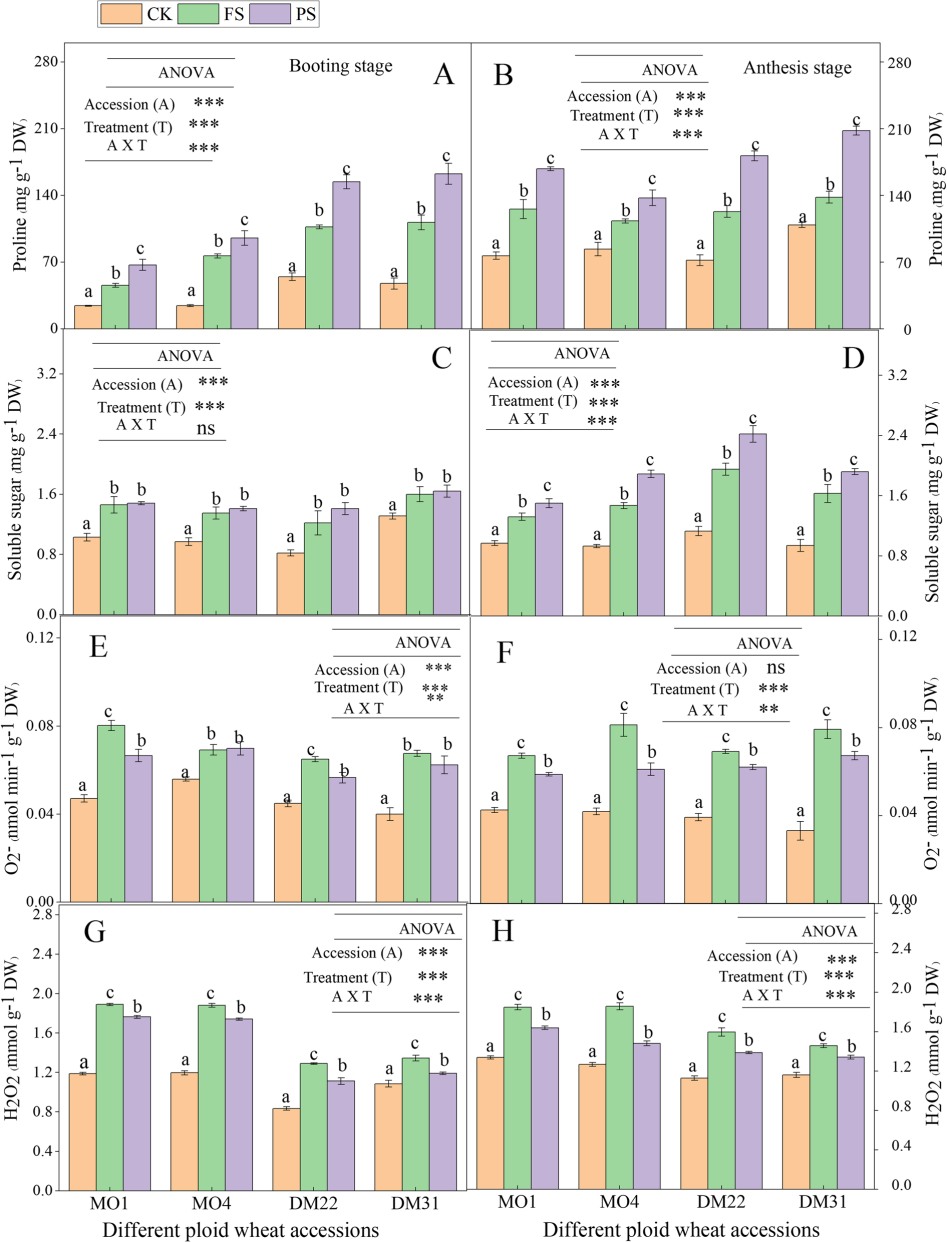
**A-H** Leaf proline content, soluble sugar and ROS under the operation of nHRS in different ploidy (diploid, tetraploid) wheat accession at booting and anthesis stage. Leaf proline content at booting stage (**A**), leaf proline content at anthesis stage (**B**), leaf soluble sugar at booting stage (**C**), leaf soluble sugar at anthesis stage (**D**), leaf O_2_^−^ production at booting stage (**E**), leaf O_2_^−^ production at anthesis stage (**F**), leaf H_2_O_2_ production at booting stage (**G**), and leaf H_2_O_2_ production at anthesis stage (**H**), in diploid and tetraploid wheat species in response to nHRS (non-hydraulic root source signaling) conditions

Leaves ROS in the form of O_2_^−^ and H_2_O_2_ were higher in PS and FS individuals than those of the CK controls and increased linearly from booting to anthesis as the soil drying period became longer of all wheat species (Fig. 3). However, under half drying root system, O_2_^−^ and H_2_O_2_ were significantly (*P* < 0.05) reduced than its FS controls, thereby declining the oxidative damage to membranes. Moreover, MO4 and DM31 showed a similar trend and did not significantly differ between FS and PS at the booting stage (Fig. 3E).

The average leaf proline concentration in PS plants relative to the average of its FS controls and FS controls were also higher than those of its CK controls during soil drying in all species, and a greater amount of proline was observed in *T. dicoccum* (Fig. 3A, B). Moreover, at the booting stage in diploid species, the proline accumulation was lower, but at the anthesis stage, both wheat species showed higher proline accumulation, particularly in PS treatments (Fig. 3A, B).

nHRS increased the soluble sugar concentration in all accessions. At booting, the soluble sugar of PS was mostly similar to the FS controls, while at anthesis, it was significantly higher in PS plants than of FS and CK controls under drought (Fig. 3C, D). Overall, the concentration of proline and soluble sugar increased from booting to anthesis with a progressively drying period among all accessions (Fig. 3).

### nHRS increased antioxidant enzyme activities in all wheat species

**Fig. 4:**
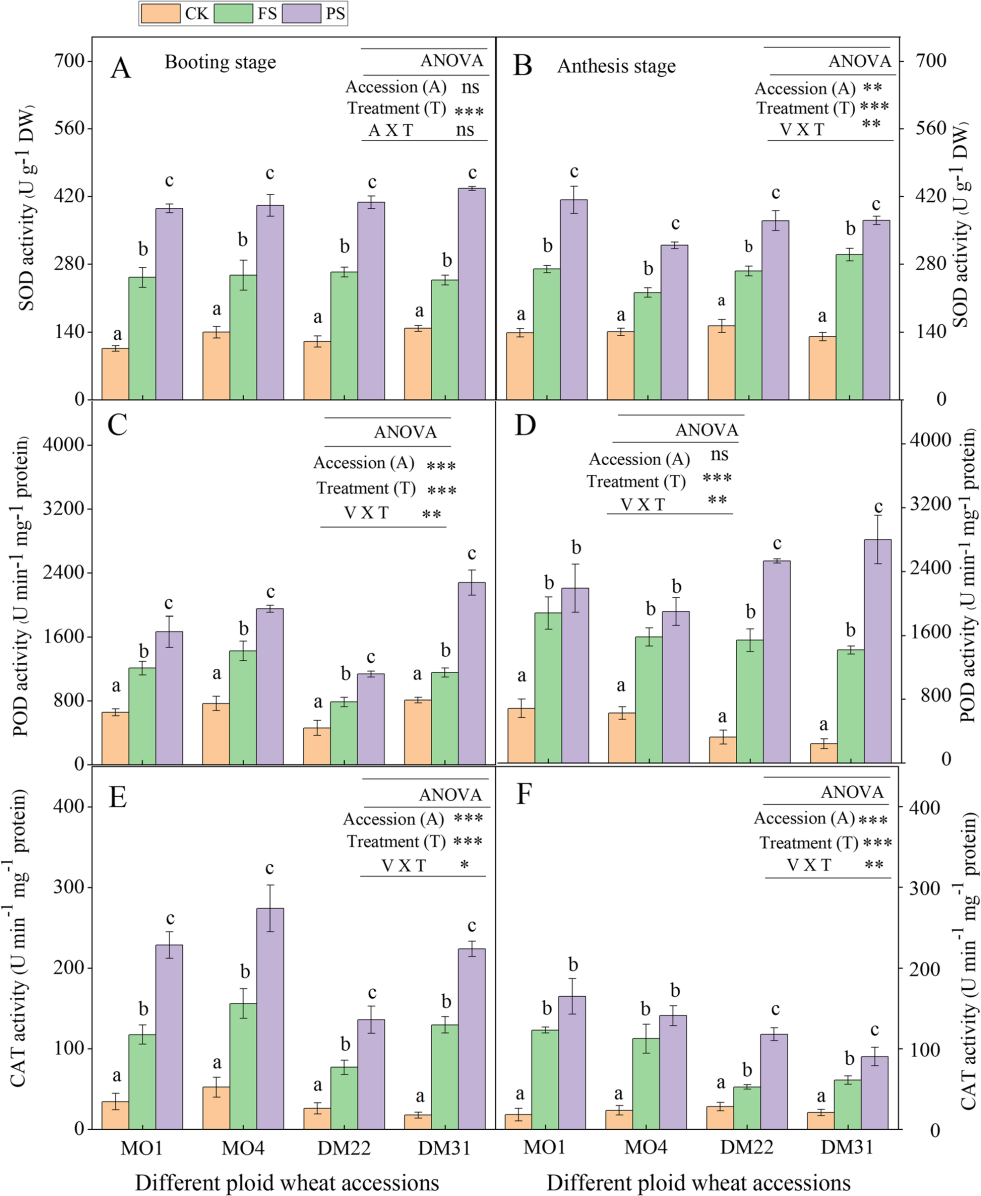
**A-F** Leaf antioxidant enzyme activities under the operation of nHRS in different ploidy (diploid, tetraploid) wheat accession at booting and anthesis stage. Leaf SOD (superoxide dismutase) activity at booting stage (**A**), leaf SOD activity at anthesis stage (**B**), leaf POD (peroxidase) activity at booting stage (**C**), leaf POD activity at anthesis stage (**D**), leaf CAT (catalase) activity at booting stage (**E**), and leaf CAT activity at anthesis stage (**F**), in diploid and tetraploid wheat species in response to nHRS (non-hydraulic root source signaling) conditions

Drought-induced antioxidant enzyme activities increased across all wheat accessions at booting and anthesis (Fig. 4). In PS plants of all species, SOD activity was significantly greater than its FS and CK controls (Fig. 4A, B) at both developmental stages. POD and SOD activity of PS plants differed among species and accessions of each specie (Fig. 4A, B, C, D). PS individuals’ POD and CAT enzyme activities were higher than FS and CK controls at the booting and anthesis stages. In contrast, PS plants’ POD and CAT activity were mostly similar to FS control in *T. monococcum* at the anthesis stage. These fluctuations among treatments were not consistent from booting to anthesis and thus were probably due to the variations and cross-talk of various root-operated signals (Fig. 4).

### nHRS declined water consumption without affecting the reproductive output

Non-hydraulic root-sourced signals significantly reduced above-ground biomass, root biomass, grain number, and grain yield in all wheat accessions (Table 1; Table 2; Table S1), especially in PS plants, whereas energy distribution varied among wheat genotypes. In CK, the grain yield increased from diploid to tetraploid species, but there were no significant differences between FS and PS in primitive wheat (Table 1). Individual size, including total biomass and leaf area, tended to increase significantly from diploid to tetraploid wheat, following a similar trend as grain yield under the regulation of nHRS. 1000-kernel weight (TKW) increased in PS of both primitive wheat species but was reduced in FS treatment across all accessions and was the highest in DM31 in PS among all the treatments and accessions (Table 2).

**Table 2:** Effects of nHRS on yield and yield components in primitive wheat

Cultivars	Treatment	Ear length (cm)	Seeds No	Spikelet No	HI	Biomass density (mg/cm^3^)	Specific leaf area (SLA (cm^2/^g)	TKW
	CK	4.58 ± 0.08b	43.2 ± 4.18b	54.0 ± 5.41a	0.29 ± 0.01a	0.89 ± 0.04a	261.05 ± 6.58a	22.75 ± 0.89b
MO1	FS	4.62 ± 0.09b	42.1 ± 3.03b	52.6 ± 3.48a	0.27 ± 0.01a	0.92 ± 0.05a	253.98 ± 6.46a	18.68 ± 0.82a
	PS	4.33 ± 0.09a	30.15 ± 3.54a	41.8 ± 4.28a	0.30 ± 0.01a	0.99 ± 0.03a	252.63 ± 13.00a	23.47 ± 0.44b
	CK	4.73 ± 0.09b	48.95 ± 4.81b	57.6 ± 5.73b	0.28 ± 0.01a	0.77 ± 0.03a	256.48 ± 14.68ab	21.94 ± 0.89a
MO4	FS	4.54 ± 0.11ab	43.6 ± 5.04b	51.9 ± 4.97b	0.26 ± 0.01a	1.03 ± 0.05b	239.86 ± 9.68a	19.65 ± 0.77a
	PS	4.26 ± 0.12a	25.55 ± 2.36a	33.8 ± 3.28a	0.25 ± 0.01a	1.26 ± 0.08c	273.24 ± 6.20b	21.23 ± 0.92a
	CK	6.23 ± 0.14a	58.55 ± 4.64b	30.5 ± 1.87a	0.46 ± 0.01b	0.73 ± 0.07a	290.65 ± 10.66a	42.91 ± 2.05a
DM22	FS	6.63 ± 0.20a	54.65 ± 3.66b	31.6 ± 1.71a	0.43 ± 0.01ab	0.81 ± 0.05ab	274.42 ± 16.61a	39.86 ± 1.46a
	PS	6.42 ± 0.18a	42.9 ± 3.05a	26.6 ± 1.98a	0.42 ± 0.01a	1.11 ± 0.18b	254.70 ± 31.81a	41.35 ± 0.27a
	CK	7.11 ± 0.16b	60.65 ± 7.14b	34.5 ± 3.95b	0.37 ± 0.01a	1.04 ± 0.17a	211.87 ± 19.17a	38.09 ± 1.18a
DM31	FS	6.75 ± 0.14ab	33.1 ± 3.94a	20.2 ± 2.32a	0.35 ± 0.03a	1.06 ± 0.10a	212.17 ± 17.56a	37.29 ± 1.28a
	PS	6.62 ± 0.12a	31.4 ± 3.51a	19.6 ± 2.15a	0.37 ± 0.03a	1.06 ± 0.08a	184.00 ± 13.49a	42.36 ± 4.96a
Accession (A)		***	**	***	***	NS	***	***
Treatment (T)		*	***	**	NS	**	NS	*
V × T		NS	NS	NS	NS	NS	NS	NS

Values are represented as means ± S. E. of the mean. Different letters within one cultivar for three different treatments at *P* < 0.05

*CK* Well water, *FS* Full root zone water stress, *PS* Partial root zone water stress

Morphological parameters reflected the changes in energy allocation across primitive wheat species under nHRS. Water deficit reduced plant height in all accessions except DM31, where plant height was higher in PS treatment (Table S1). The reduction rate in grain number was higher in PS plants than in FS and CK of all wheat species (Table 2). Also, two parameters related to resource acquisition efficiency, specific leaf area (SLA) and biomass density (the rate of above-ground biomass over canopy volume) showed a contrasting trend in diploid and tetraploid wheat species. SLA increased in PS of MO4 plants; generally, the CK and FS plants did not have a significant difference for SLA across the wheat species. Overall, biomass density increased significantly in FS and PS in MO4 plants and was highest in PS (Table 2). Soil drying treatments reduced the water consumption in three wheat species, whereas the reduction in water consumption was highest in PS individuals (Table 1). WUE_G_ increased in PS plants to those of FS and CK controls, and the average relative increase was found in tetraploid species. The reduction in water consumption in PS plants may not affect grain yield but might increase WUE_G_.

**Fig. 5:**
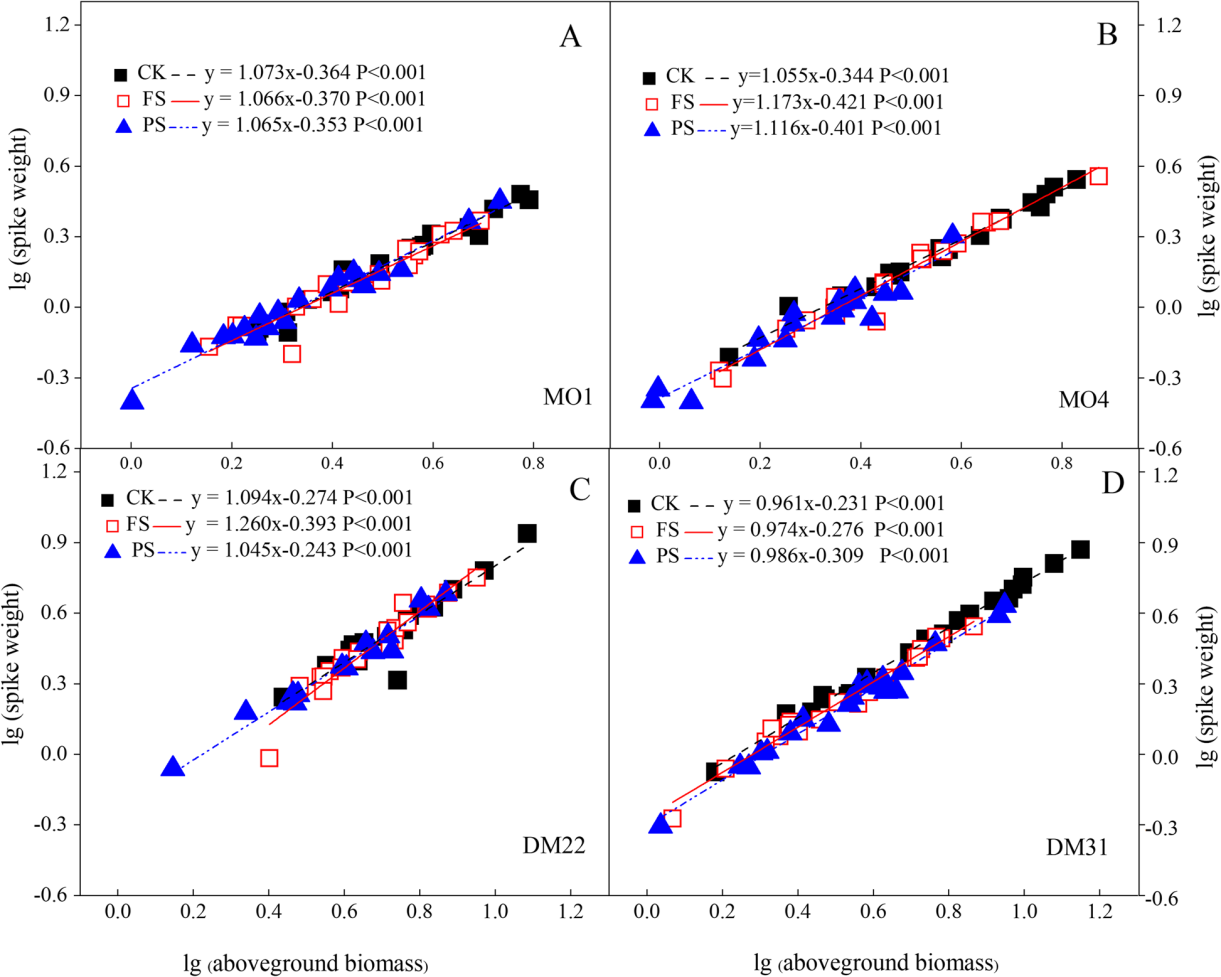
**A-D** Allometric relationship between spike weight and above-ground biomass (Bivariate plots of log-transformed spike biomass vs aboveground biomass), among water treatments in diploid and tetraploid wheat species. The black dotted lines are the regression lines of well-watered treatment; the red solid lines are the regression lines of nHRS treatment for FS, and the blue small dotted lines are the regression lines of nHRS treatment for PS (same in the below). All equations were significantly fitted (*P* < 0.001) (same in the below). **A**, and **B** represent the bivariate plots in diploid primitive wheat. **C**, **D** shows the bivariate plots in tetraploid primitive wheat

To compare the differences in reproductive allocation between the primitive wheat types, bivariate plots were used to evaluate the exponential relationships between spike weight/ear biomass vs above-ground biomass (log-transformed) (Fig. 5). In general, and there existed significant quadratic power functional relationships in three groups of variables (*P* < 0.01). In primitive wheat, there was a significant allometric relationship between ear biomass and above-ground (log-transformed), and the value of α (slope) was significantly more than 1 due to nHRS regulation. However, in the DM31 wheat genotype, no significant allometric relationships were observed. In contrast, there existed an isometric R–V relationship in this wheat genotype since the value of α was 1.038 and 0.978 in CK and nHRS groups, respectively, not significantly different from 1 (Fig. 5D). This result indicated an isometric relationship between the three variables in DM31, a primitive wheat (Fig. 5).

## Discussion

Non-hydraulic, root-to-shoot signaling of soil drying is a comparatively modern hypothesis concerning maintaining plant leaf water status since ABA is considered one of the keys for long-distance signaling. Previously it was considered that drying soil first might affect the foliage as it caused a lower water status in the shoot. Still, reduced *gs* can maintain the leaf water status under soil drying, and this stomatal closure might be induced by a high accumulation of ABA in the plant [33]. Thus, it appears that ABA, a non-hydraulic material, enables the plants to ‘sense’ drying soil by roots and act as an early warning response to the aboveground plant parts by closing the stomata [34]. The logical origination of this mechanism to sense the drying soil by roots is widely explored, and different signaling pathways might involve that interplay together to perform a signaling cross-talk.

**Fig. 6:**
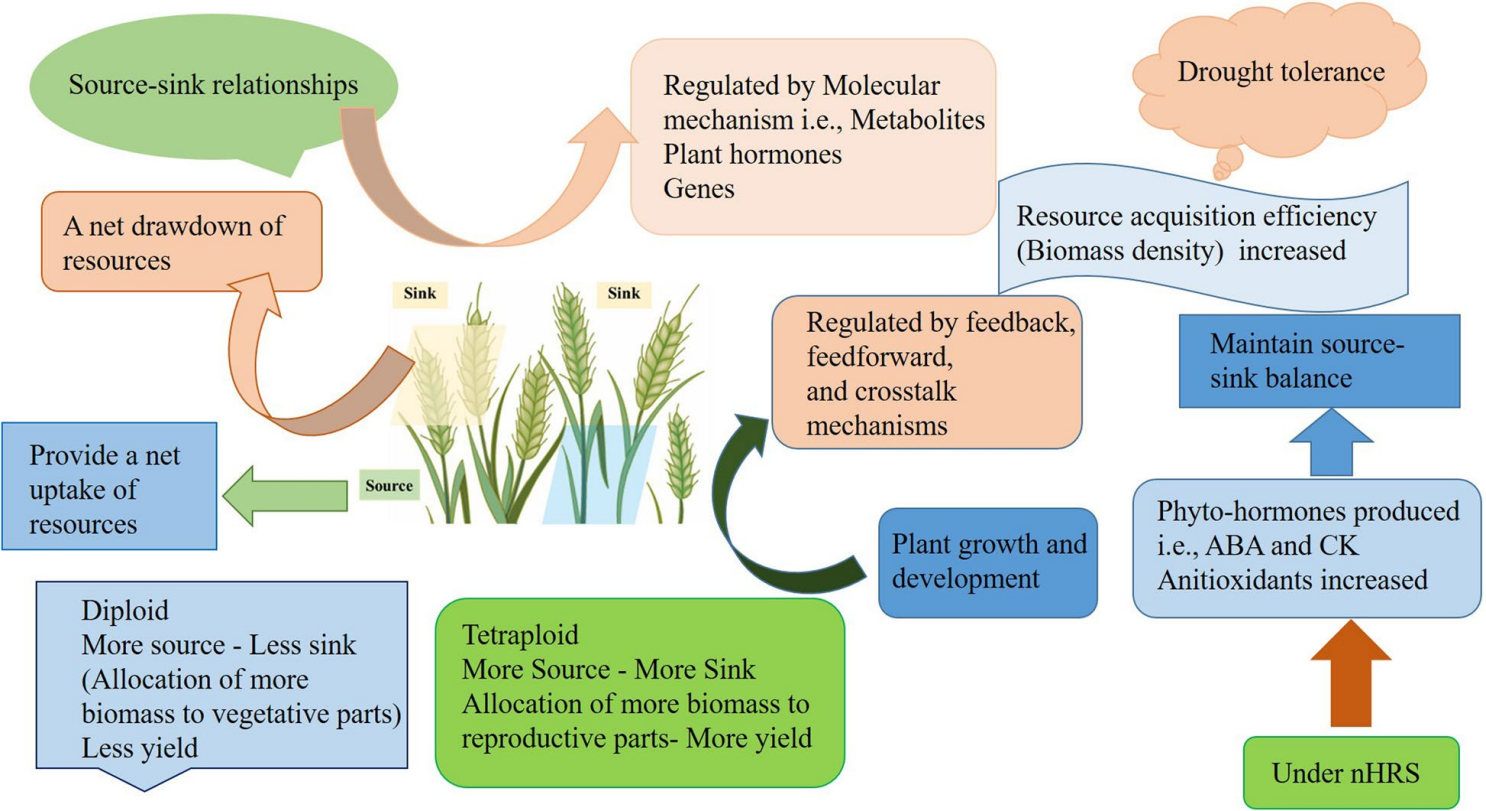
Schematic diagram of plant processes under nHRS in different ploidy (diploid, tetraploid) wheat accession. Here nHRS represents non-hydraulic root to shoot signaling, ABA Abscisic acid and CK represent cytokinin

In the present study, one-half of the root system of PS wheat plants was watered. At the same time, the other half was dried throughout the drying period, and the water status (measured in terms of LWP and LRWC) of these plants was similar to both holistic root treatment and fully watered controls. The similar leaf water status across treatments of all plants suggests that a significant decline in stomatal conductance of split root system plants might be due to chemical signals such as higher concentrations of ABA (Fig. 1, 2, 6). LWP and LRWC have been used as reliable indicators to study the nHRS signal production in plants [4, 35].

Various studies comparing PS relative to FS foliage have found much stronger evidence for the nature of the nHRS signal. Different components involved in root-to-shoot signaling might fluctuate quickly and extensively with the change in environmental conditions [5]. Probably, the decline observed in *gs* and greater accumulation in ABA of PS plants relative to CK plants could have been due to the unmeasurable plant physio-chemical status. Therefore, using only CK control (receiving nearly twice as much water as the PS plants) would have had two drawbacks: (1) the measurement of different attributes might have small fluctuations and were not sensitive enough, and (2) measurements of ABA, CK, and other physio-biochemical parameters were not continuous, occasional and invasive, thus going through temporary changes in ABA concentration and leaf water status between PS and CK plants. The purpose of using FS individuals was to overlook the possible undetected effects of ABA accumulation caused by the declining water availability in PS plants to half, compared to CK plants. Half-dried (PS) and FS plants had received a similar amount of water throughout the drying period, so greater accumulation in ABA of PS plants compared to FS would have been due to the nHRS signal. Moreover, severing half of the root system somewhat enhanced ABA concentration across all wheat species.

Though the role of ABA as nHRS signaling material is now fairly well recognized for different ploidy wheat accessions [36], its importance as signaling cross-talk with other components in different ploidy wheat accessions has been questioned. An increase in ABA concentration was directly related to a decline in gs in wheat plants [37]. This observation might lead to the conclusion that wheat plants would be more likely to use ABA as nHRS to control *gs*. Wheat plants generally have high transpiration and photosynthetic rates, and especially primitive wheat has an extensive and deep root system to absorb a large amount of water. These factors might reduce the impact of drought stress; therefore, the morpho-physiological status of different ploid wheat species may depend on environmental variation [38]. However, due to the decline in gas exchange across all wheat species, stomatal behavior is expected to be regulated much, typically controlled by drought-induced ABA accumulation [39]. As described above, high root system wheat species might display relative physiochemical homeostasis under nHRS however, tetraploid wheat species, with a small root system, exhibited adverse growth responses in PS treatment, but the plants’ growth was much better in FS, which suggests that holistic root system of FS plants might more closely tied water deficit adaptation and nHRS signaling.

Much of the work on non-hydraulic signaling under drying soil has been conducted in various plant species, including wheat [4, 36]. Other than ABA, another root-derived signal could have been involved, such as a change in pH or the concentration of other phytohormones [36]. Cytokinins are recognized to maintain the plant responses to drying soil [12, 40]. However, zeatin riboside (ZR) and isopentenyl adenine (iPA) are the predominant types of cytokinins (CK) present in plants that are related to plant stress adaptive strategies [41]. In our study, a decline in ZR concentration in leaves was observed, which contradicts the study of Merewitz, (2010), who found an endogenous increase in ZR and iPA content in leaves of transgenic creeping bentgrass under water deficit [42]. However, it agrees with Batool et al.’s (2019b) observation that CK and ABA have signal cross-talk under drought and work antagonistically to induce stomatal closure and hold the leaf water status [43].

Numerous factors, including as growth rate, stress intensity, genotype, length of stress, photosynthetic machinery activity, respiration, transpiration, and ambient circumstances, all affect how plants react to drought. Many genes in wheat plants, are implicated in drought stress tolerance and produce a variety of enzymes and proteins i.e., rubisco, proline, helicase, late embryogenesis abundant (LEA), responsive to abscisic acid (Rab), glutathione-S-transferase (GST), and carbohydrates (). Three well-established methods exist for managing drought stress: avoidance, escape, and tolerance. Plants that are tolerant of drought might lessen its negative effects. The ability of plants to provide a high water potential with decreased soil hydrologic supply and, in reality, prevent dehydration, is what allows them to avoid drought. Plants that are capable of withstanding minimal water injury and internal water shortages are said to be tolerant of dehydration. Leaving is another strategy to deal with the drought. Long before a drought occurs, this is where the plant completes its life cycle.In the present study, increased activity of osmoprotectants (proline and soluble sugar) confirms that enhanced ABA concentration through nHRS signaling in primitive wheat accessions maintained the cell/plant adaptability. Hence, drought stress significantly increased the O_2_^−^ and H_2_O_2_ production (Fig. 3), suggesting that increasing ABA accumulation in the wheat plants might have enhanced ROS generation and mitigated the cellular membrane damage under drying soil [43]. Our results are similar to the findings of Jiang and Zhang, (2002), who revealed that ABA can trigger the ROS generation in the seedlings of maize plants [44]. Our study is one of the first to exhibit enhancing ABA production could protect plants from water deficit-induced oxidative damage and regulate better drought tolerance by suppressing ROS due to the ABA activation of an antioxidant defense system [44]. Various antioxidant enzymes, including SOD, POD, and CAT, were increased in concert with ROS (Fig. 4) might have mitigated the ROS effects. SOD encompasses enzymes for the dismutation of O_2_^−^ to H_2_O_2,_ while CAT and POD reduce H_2_O_2_ [45]. While there was a significant increase in SOD, POD, and CAT levels of PS plants than its FS and CK controls suggesting that enhanced ABA of PS plants may be activated SOD level to transform O_2_^−^ to H_2_O_2_ and then removal and conversion of this H_2_O_2_ to H_2_O through POD and CAT across different ploidy wheat accessions. Our results are similar to other studies suggesting that the upregulation of antioxidant enzyme genes has been associated with enhancing antioxidant enzyme activities [46].

One of the main obstacles to wheat production is drought, which is becoming a more significant issue in many of the world’s wheat-growing countries [47]. The growth and productivity of the wheat is severely reduced as a result. Later phases of wheat crop under water stress can result to a reduction in the weight and quantity of kernels per ear [48]. Although losses can occur at any point in the crop’s growth stages, however during anthesis stage, drought stress has the biggest effect on yield reduction. Grain production reduces when water stress occurs during anthesis because it reduces pollination, which leads to fewer grains being produced per spike. Sufficient water during or after anthesis allows the plant to increase the rate at which it synthesises photosynthetic energy and gives it more time to transfer carbohydrates into grains, which leads to larger grains and increased grain yield [49]. Drought reduces radiation utilization efficiency, which in turn lowers growth rate during important growth phases such tillering, booting, earing, anthesis, and grain development stages. The crop’s performance throughout these crucial phases is determined by the availability of water [50].

In present study nHRS decreased the total water consumption while improving the WUE_G_ but did not enhance the grain yield in all wheat species in both FS and PS treatments (Table 2). Thus, ABA signaling as nHRS material improved the desiccation tolerance of all wheat accessions. However, this did not significantly influence its drought resistance ability, defined as yield under a water deficit environment. Under drought conditions, high accumulation of ABA did not increase the grain yield. Our finding is surprising in the light of a study by Trvaglia et al., (2010), who observed an increase in wheat grain yield by spraying ABA to the leaves [51]. A possible explanation could be that higher ABA production in PS plants was not high enough to affect plant yield.

An essential physiological process for figuring out how much dry matter accumulates in crops is the source-sink relationship. The terms “source” and “sink” denote the potential capacities for photosynthesis and storage and metabolism of the products of photosynthetic processes, respectively. The primary source for wheat plants is the leaf, which is essential for the development of the grain as well as other components like the stem and sheath. The term “sink” pertains to the newly formed tissues and grains, which can be further classified as either metabolic or storage sinks. When wheat reaches its late growth stage, grain makes up the majority of the crop. Thus, the primary variables that could restrict grain yield are the photosynthetic activity of leaves (source) and the ability of grains to store following anthesis (sink). The two primary mechanisms influencing wheat yield are the generation of leaf assimilates and their use in seed development [52]. The chief purpose for comparing and characterizing the physio-biochemical and morphological sensitivity of these four wheat accessions to nHRS of soil drying was to explore the source-sink relations and metabolic rate connecting other stress signals in diploid and tetraploid (primitive) wheat. Is relatively different ploidy wheat species’ sensitivity to nHRS allied with other biochemical and physiological tendencies that characterize plants as drought-avoidant or tolerant? Particularly, do the wheat species generally characterized as drought avoiders have higher nHRS induce signaling to soil drying than drought tolerant species? Previous studies showed that modern wheat is more drought-tolerant than primitive and further research is needed in this context [53].

## Conclusion

It is essential to investigate the tradeoff between source and sink for plants under the active defense to drought stress, such as nHRS. The present study was performed to study the role of non-hydraulic root source signaling (nHRS) on the production of phytohormones and their crosstalk with other stress signals in two growth stages of wheat. Photosynthetic activity, source-sink relations and yield formation were observed under nHRS in diploid and tetraploid wheat species. Manipulations of source and sink balance were maintained by root-to-shoot signaling under drought stress. Diploid and tetraploid wheat showed variation in responses for phytohormones accumulation and antioxidant defense mechanisms at two growth stages. Overall, the booting stage appeared to be more sensitive to nHRS-induced phytohormones accumulations and their signaling crosstalk with other stress signals than the flowering stage. Further research is needed to unravel the precise molecular mechanisms underlying these interactions and to enhance stress tolerance in crops.

## Materials and methods

### Plant materials and growth conditions

Two relatively independent but closely related pot-culture trials were conducted from March to August 2015 at the Yuzhong Experiment Station of Lanzhou University, Yuzhong County, Gansu Province (35^o^51'N, 104^o^07' E, altitude 1620 m), northwest China. Two wheat species, i.e., four accessions, including two diploids (*Triticum monococcum*) MO1 and MO4, and two tetraploids (*Triticum dicoccum* Schuebl.) DM22 and DM31 were selected as study materials. DM22 is referred to as a premium genotype among the primitive wheat species. According to our previous observations, the same species had a similar phenological cycle, and different species were found to have different growth periods [24, 54]. The Institute of Crop Germplasm Resources, Chinese Academy of Agricultural Sciences, Beijing, China, provided seed resources for diploid and tetraploid wheat. The accessions were grown in a rainout shelter (50m long × 24cm wide × 5.7 m high) that can be managed for opening and closing according to the weather forecast.

Wheat seeds were surface-sterilized with 0.2% (w/v) HgCl_2_ for 600 s, then washed with distilled water and vernalized at 4 °C for 1 day. They were kept on moistened filter paper with distilled water in the dark for germination in an incubation cabinet at 25 °C. Uniform fourteen seeds were sown in each of 144 plastic pots (250 mm diameter × 380 mm high) with equal spacing between the seedlings. Pots were filled with 11 kg of sieved loess soil-based substrate (loess soil: vermiculite, v/v = 2:1). Before sowing the seeds, two chemicals, NH_4_NO_3_ and KH_2_PO_4,_ were added as a fertilizer in each pot with final concentrations (µg g-1 dry soil): P 22.1, K 27.9, N 188. Soil water content (SWC) at the field was determined by watering to excess and then allowing the pots to drain until 2 days before weighing. After two weeks of germination seedling were thinned to keep 9 plants per pot for both trials. After the emergence of seeds, all the plants were watered daily after weighing and maintaining the 90% FC and were allowed to grow before water stress initiation. Though the date of sowing for all cultivars was the same, water stress was applied according to their respective developmental stages to attain synchronization in the experiment according to their growth circle.

### Experimental design, nHRS treatments and sampling at booting and anthesis stages

A split-root trial was conducted to expose the cross-talk characteristic of major root-sourced chemical signals and their interplay with other drought-stressed signals in different ploidy wheat species. Two diploid varieties, MO1 and MO4, have the longest growth cycle, about 10 to 25 days longer than tetraploid cultivars (DM22 and DM31). Three water treatments were designed by using different methods for plant growth. Water treatments were exposed at the jointing stage, including 1) the Control group with 90% field water capacity (FWC) maintenance throughout the growing period (CK); 2) the Intact root system drying group (FS group) with 55% FC to induce nHRS and 3) 70–45% Split-root treatment group (PS group) with alternative water supply (half wet and another half drying by splitting the roots) in two parts of the root system. Split-root treatment was designed as watering half root system (70%-45% FWC) and the remaining half system drying subsequently. In this group, each pot was allowed to be dried until around 45% FC in one half, while the other half was rewatered to 70% FC. A divider was placed in the middle of each pot to prevent substance exchange between the two parts [34]. Soil media was equally filled in halves of each pot, and seeds were sown at the boundary above the divider. Following two days of treatment (according to preliminary observations), the two halves were drying and wetting alternatively. In drought stress treatment with the intact root system, each pot was maintained at nearly 55% FWC in soil moisture, in which soil water content (SWC) fluctuated between 70–45%. Harvest was taken at each accession’s booting and flowering stage according to their developmental period since imposing the drought stress at the jointing stage. To measure different biochemical and physiological attributes, fully expanded leaves were collected, four pots per treatment per wheat accession, and immediately frozen in liquid nitrogen. Leaf gas exchange characteristics were measured between 9:00 am and 11:00 am, while LRWC and LWP were measured mid-day.

Root samples were taken and washed in all drought treatments for biomass determination. Roots were separately sampled and determined in each half of the pot in the split-root treatment group. In this study, SWC was measured gravimetrically by weighing the pots and expressed as a percentage of available water with FWC. Water treatments started at the jointing stage according to the developmental stage of each accession. For each wheat species/accession, considering their respective developmental stage by combining treatment, 12 pots were used to get the harvest, with each variety replicated four times.

After imposing water treatments, SWC (measured gravimetrically by weighing the pots and expressed as a percentage of available water with FWC) was measured daily. In contrast, leaf relative water content (RWC), and gas exchange characteristics, including stomatal conductance (*gs*), photosynthetic rate (*Pn*), and transpiration rate (*E*), were measured for each pot at booting and flowering stages, respectively. A completely randomized design was used for four different ploidy wheat cultivars. Each measurement had four replicates by selecting the upper fully expanded leaf (the 2nd leaf from the top for LRW and LWP while the 1st leaf from the top for gas exchange characteristics. Stomatal conductance for each replicate was the mean of five readings for each leaf measured between 9:00 am and 10:00 am by using an LI-6400 portable photosynthesis system (Li-Cor, Lincoln, NE, USA) following the recommended measuring precautions [55]. Determination of signal cross-talk for the four wheat species under non-hydraulic root signal was observed by collecting the leaf samples for different analyses. Leaf samples were collected at the booting and anthesis stage for measuring different physiological parameters with four replicates for each treatment, so 48 pots were used at each sampling time. Water stress treatments were continued for the remaining 48 pots until the final harvest.

The SWC of water-stressed pots was measured by daily weighing during the whole growth period of wheat plants. By using the following formula, SWC was calculated:

$$\mathrm{SWC }= ({\text{Wt}}-{\text{Wd}}-{\text{We}}-{\text{Wp}}) / (\mathrm{Wd }\times \mathrm{ FWC}) \times 100\mathrm{\%}$$

Wt represented the temporary whole pot weight when we collected fresh leaf samples and measured gas exchange attributes. We are the weight of the empty pot, Wp is the estimated fresh weight of all plants in the pot, Wd is the dry soil weight, and FWC is the field water capacity [4]. Plant height, length, and width per leaf were measured with a ruler to the nearest millimetre. A sum of all individual leaf areas was the total leaf area, and the following method was used to calculate individual leaf area:

$$\mathrm{Leaf\,area }=\mathrm{ leaf\,length }\times \mathrm{ maximum\,leaf\,width }\times 0.73$$

The value, 0.73, was predetermined and constant by measuring the leaf area for a range of plants with a scanner (Epson 10,000 XL, Epson Canada Ltd, Toronto, Ontario), the leaves length, and maximum width on the plant with the ruler.

Two third of the leaf discs (5 mm in diameter) were used to measure fresh weight (FW) by sampling and weighing immediately. The discs were kept in the tubes having fresh distilled water for 8 h under 10 μmol m^−2^ s^−1^ PPFD, and instantly dried using filter paper, weighed measured the SW (saturated weight) and subsequently dried it for 24 h at 80 °C in a forced-draught oven. Leaf RWC was calculated as RWC = ((FW-DW)/(SW – DW)) × 100 [56], SW is saturated weight, DW is dry weight, and FW is the fresh weight.

### Source-sink relations measurement

To determine the role of ABA as nHRS material on yield and yield components and its effect on source-sink relations of two wheat species (four different ploid accessions, two diploid and two tetraploid) under drought, three drought-stressed treatments were subjected from the jointing stage of each accession. The water deficit treatment was imposed by withholding the water until the soil water content (SWC) reached the predetermined level: (i) 16 pots were maintained at about 90% FC by giving water daily in the evening before sunset; (ii) 16 pots, used split root design by imposing the divider, were allowed to dry until the SWC reached to 45% the total weight of the pot and watered on one part to 70%, the cycle was maintained and let pots to dry again at 45% FC and rewatered from the other part to 70% FC; and (iii) 16 pots, the same method for imposing water as the treatment (ii) but divider was not used to separate the roots, each treatment per accession combination was used in four time replication.

Three drought treatments to measure the root to shoot signaling were maintained until the maturity of diploid and tetrploid wheat accessions. At the physiological maturity (~ 110 DAS), whole plants were harvested as defined by the complete disappearance of the glumes’ green colour. Plant roots were washed free of soil by using a screen (0.4 mm). At maturity stage, plant height, fertile spikelet number, yield, and yield components per plant were recorded, then divided into shoots (including leaves and husks), grain, and roots after drying for 48 h at 80°C and then weighed with a digital balance. Data for water use were collected by recording the daily water added during the whole plant’s life. Water use efficiency was determined by WUE_G_ (water use efficiency for grain) = grain weight/ total water used from sowing until harvest. Under each treatment other variables were calculated, such as harvest index (HI) = grain yield/above-ground biomass; biomass density = above-ground biomass/ (plant height × leaf area); specific leaf area (SLA) = leaf area/leaf biomass.

### Determination of Leaf free proline and soluble sugar

Free proline was determined by following the method of Bates et al. [57]. The frozen leaf segments (0.5 g) were grounded by a mortar with liquid nitrogen and homogenized in centrifuge tubes with 5 mL phosphate buffer (pH 7.8; 0.5 M). After centrifugation (15,000 rpm for 0.25 h at 4°C), the supernatant was obtained and used for further biochemical analyses, and absorbance was recorded at 520 nm. Soluble sugar concentration was measured using the anthrone reagent method [15].

### Measurements of Enzyme assays

Frozen leaf parts (0.5 g) were ground and homogenized in 50 mM phosphate buffer (pH 7.8) under liquid N_2_ with a mortar and pestle. The homogenate was centrifuged at 15,000 g for 15 min at 4 °C, and the supernatant was used to determine the following key enzyme activities.

### Measurements of Total superoxide dismutase (SOD)

SOD activity was determined by following the method of Batool et al. [34] by observing the inhibition of photochemical reduction of NBT nitro blue tetrazolium. The 3ml reaction mixture consists of 13mM methionine, 50Mm potassium phosphate buffer (PH 7.8), 75 µM NBT, 0.1 Mm EDTA, 100µl enzyme extract and 2µM riboflavin. Reaction mixtures were lightened for 900 s at 100 µmol m^−2^ s^−1^ light intensity. The amount of enzyme needed for causing 50% inhibition for NBT reduction is defined as one unit of SOD activity monitored at 560 nm.

### Measurements of Catalase (CAT)

CAT activity was determined by the H_2_O_2_ disappearance (coefficient of extinction 39.4 Mm^−1^ cm^−1^) for 110 s at 240 nm [43]. 3 ml reaction solution was comprised of 50mM potassium phosphate buffer (pH 7.0), 20 µl enzyme extract, and 10 mM H_2_O_2_.

### Measurements of Peroxidase

Peroxidase activity was determined for 100 s at 470 nm. In the reaction mixture, 3 ml guaiacol solution, 10µl enzyme extract, and 10 µl 30% H_2_O_2_ were added.

### Measurements of ROS (reactive oxygen species)

O_2_- production was determined using nitrite formation from hydroxylamine in O_2_^−^ presence [34]. The leaf sample (0.5 g) was crushed and homogenized by 5ml 50 mM potassium phosphate (pH 7.8), then centrifuged at 5000 g for 600 s at 4 °C. The reaction mixture contained 1 ml supernatant and 1 ml of 1 mM hydroxylamine hydrochloride (50 mM phosphate buffer (pH 7.8) used as solvent). After incubating at 25 °C for 1200 s, 7 mM α-naphthylamine and 17 mM sulphanilamide were added. The absorbance was read at 530 nm after the reaction at 25 °C for 1200 s. H_2_O_2_ was determined by observing the titanium-peroxide complex absorbance at 415 nm [58], which was calculated using a known H_2_O_2_ concentrations standard curve.

### Abscisic acid (ABA) and cytokinin (CK) extraction, purification, and quantification

Extraction and purification methods for abscisic acid and cytokinin determination were modified from those given by Du and Batool [43, 59]. Plant leaf segments were grounded in liquid N_2_ with a mortar and pestle, extracted using pre-cooled 80% methanol (v/v) containing 1mM butylated hydroxytoluence to inhibit oxidation, and then stored overnight at 4 °C. The next day, plant extracts were centrifuged at 1000 g for 900 s at 4 °C. Residues were mixed in the same pre-cooled extraction solution and kept for 1 h at 4 °C, and samples were centrifuged again at 4 °C for 900 s at 10,000 g. Supernatants, after combining, were passed through Chromosep C18 columns and prewashed with 5 ml of 80% and 10 ml of 100% methanol. Collected efflux was dried using nitrogen for evaporation. 1.6 ml phosphate-buffered saline (PBS) comprising 0.1% (w/v) gelatin (pH 7.5) and 0.1% (v/v) Tween 20 were used to dissolve the residues for analysis by ELISA (enzyme-linked immunosorbent assay).

Antigen and antibody of mouse monoclonal against ABA and CK and IgG-HRP (immunoglobulin G-horse radish peroxidase were used in ELISA. The ABA quantification method by ELISA has already been described [60]. In the present study, each hormone percentage recovery was calculated by adding known amounts of standard hormone in a split extract. According to earlier studies, monoclonal specificity was confirmed, and the possibility of other non-specific inhibitors was excluded [60].

### Statistical analysis

Data is presented as means of 4 replicate samples for physiological and biochemical parameters at booting and anthesis stages, and 20 replicate samples from each pot (4 pots for each treatment) were analyzed for source-sink relations measurement. Data of measured variables were examined by one-way and two-way ANOVA (analysis of variance) (water treatments and wheat accessions). SPSS (SPSS 24.0 version, Chicago, IL) for Windows was used to conduct all the data analyses, and the means were compared by Duncan’s multiple range tests at *P* < 0.05. The standardized major axis tests and routines (SMATR) software package [61] was used to determine whether an allometric relationship existed. Origin 2021 version (Microcal Software Inc) was used to draw the figures and perform the analysis.

### Supplementary Information

**Supplementary Material 1.**

## Data Availability

The original contributions presented in the study are included in the article, and further inquiries can be directed to the corresponding author.
