# Supplementary material for: Root-to-shoot signaling positively mediates source-sink relation in late growth stages in diploid and tetraploid wheat
Source: BMC Plant Biol. 2024 Jun 3;24:492. doi: 10.1186/s12870-024-05046-z (PMC11145845; doi:10.1186/s12870-024-05046-z)
Supplement: Supplementary file 1 — Supplementary Material 1. [file 12870_2024_5046_MOESM1_ESM.docx]

**Table S1**  Effects of water stress on growth parameters in primitive wheat (diploid, tetraploid) wheat cultivars.

| Cultivars | Treatment | Total tiller number | Fertile tiller | Plant height (cm) | 1^st^ leaf area | 1^st^ leaf biomass  (g/plant) | Stem weight | Root biomass (g/plant) | Ear biomass (g/plant) | Above-ground biomass (g/plant) |
| --- | --- | --- | --- | --- | --- | --- | --- | --- | --- | --- |
|  | CK | 4.1+0.31a | 3.8+0.34 a | 64.55+1.08c | 18.11+ 0.89b | 0.07+0.01b | 1.34+0.13b | 0.59+0.03a | 1.59+0.16b | 3.36+0.32b |
| MO1 | FS | 4.1+0.20a | 3.7+0.21a | 60.96+1.32b | 12.21+1.09a | 0.05+0.01a | 1.16+0.09ab | 0.45+0.05a | 1.35+0.11ab | 2.93+0.22ab |
|  | PS | 3.7+0.27a | 3.3+0.26a | 57.23+0.94a | 10.99+0.73a | 0.05+0.01a | 0.95+0.09a | 0.54+0.09a | 1.16+0.13a | 2.45+0.25a |
|  | CK | 4.8+0.39b | 4.2+0.37b | 62.64+0.89b | 23.86+5.82b | 0.11+0.02b | 1.43+0.12b | 0.47+0.07a | 1.88+0.18b | 3.84+0.35b |
| MO4 | FS | 4.3+0.36b | 3.9+0.32b | 60.15+0.92ab | 14.03+1.68ab | 0.05+0.01a | 1.25+0.12b | 0.40+0.07a | 1.46+0.17b | 3.12+0.32b |
|  | PS | 3.2+0.25a | 2.9+0.23a | 58.25+1.28a | 8.41 + 1.57a | 0.03+0.01a | 0.93+0.07a | 0.33+0.05a | 0.93+0.08a | 2.14+0.16a |
|  | CK | 3.1+0.16b | 2.6+0.15ab | 72.68+1.69b | 42.16+2.13c | 0.15+0.01b | 1.68+0.13b | 0.64+0.08b | 3.49+0.35a | 6.03+0.62b |
| DM22 | FS | 2.9+0.11b | 2.8+0.12b | 66.35+1.43a | 29.85+3.57b | 0.13+0.01ab | 1.35+0.09a | 0.57+0.05ab | 3.05+0.26a | 4.94+0.36ab |
|  | PS | 2.5+0.14a | 2.2+0.16a | 64.76+1.51a | 19.86+2.25a | 0.10+0.01a | 1.28+0.11a | 0.42+0.02a | 2.66+0.25a | 4.33+0.36a |
|  | CK | 3.3+0.23b | 3.1+0.30b | 78.71+1.48ab | 28.01+4.45a | 0.13+0.02b | 2.16+0.29b | 0.40+0.03a | 3.43+0.42b | 6.31+0.79b |
| DM31 | FS | 2.3+0.19a | 2.0+0.22a | 75.89+1.62a | 15.22+2.38b | 0.07+0.02a | 1.34+0.14a | 0.38+0.07a | 1.83+0.18a | 3.59+0.38a |
|  | PS | 2.1+0.11a | 1.7+0.11a | 82.13+1.59b | 11.62+ 0.81b | 0.06+0.01a | 1.52+0.20a | 0.33+0.02a | 1.83+0.22a | 3.82+0.46a |
| Accession (A) | | *** | *** | *** | *** | *** | *** | *** | *** | *** |
| Treatment (T) | | *** | *** | *** | *** | *** | *** | *** | *** | *** |
| V×T | | NS | NS | *** | NS | * | NS | NS | NS | NS |

Notes: Values are represented as means + S. E. of the mean. Different letters within one cultivar for three different treatments at P<0.05; CK; well water, FS; full root zone water stress, PS; partial root zone water stress.
